# The pattern of mucocutaneous disorders in HIV infected children attending care and treatment in Tikur Anbesa specialized hospital, Addis Ababa, Ethiopia

**DOI:** 10.1186/1471-5945-13-12

**Published:** 2013-10-25

**Authors:** Yichalal Endayehu, Amha Mekasha, Firehiwot Daba

**Affiliations:** 1 Department of Pediatrics and Child Health, College of Health Sciences, School of Medicine, Addis Ababa University, Addis Ababa, Ethiopia; 2 Department of Dermatology and Venerology, College of Health Sciences, School of Medicine, Addis Ababa University, Addis Ababa, Ethiopia; 3 PO Box 90074, Addis Ababa, Ethiopia

## Abstract

**Background:**

Children with HIV infection may develop a wide variety of infectious and inflammatory diseases of the skin. These disorders are often more severe and more difficult to treat than in the immunocompetent child. In some cases, disorders of the skin or mucous membranes may provide an early clue to the presence of pediatric HIV infection.

**Methods:**

It is a descriptive cross-sectional study which was conducted at the pediatric infectious disease unit, department of pediatrics and child health, Tikur Anbesa specialized Hospital. Clinical information was collected using a questionnaire. The data was analyzed using SSPS 16.0 version. Chi-squared was utilized where appropriate and a p-value of less than 0.05 was considered statistically significant.

**Results:**

Two hundred seventy HIV infected children were recruited in this study. Out of these females were 51.5% and males were 48.5%. Most of the children 196/270 (72.6%) were suffering from one or more mucocutanous disorders. The most prevalent mucocutanous disorders were infectious dermatosis. Overall, mucocutaneous disorders were more prevalent in advanced stages of HIV disease P < 0.001. Two or more mucocutanous disorders were found in moderate and sever immunosuppression. Seventy three percent of the HIV-infected children with mucocutaneous disorders were already on HAART.

**Conclusions:**

The prevalence of mucocutanous disorders is high in HIV infected children. Advanced immunosuppression is highly associated with a wide spectrum of mucocutanous disorders.

## Background

Globally, there were a total of 33.2 million people living with HIV, in 2007, of which 2.5 million (7.5%) were children under the age of 15 years [1]. Total number of deaths was 2.1 million, of which 330,000 were children. It is estimated that more than 90% of children living with human immunodeficiency virus (HIV) acquired the virus during pregnancy, birth, or breastfeeding, forms of HIV transmission that can be prevented [2].

In Ethiopia 44% of the population are children below 15 years. The adult prevalence of HIV is 7.7% in urban and 0.9% in rural with average population prevalence 2.1%. There are 134,586 children living with HIV/AIDS and out of whom more than 67,000 are estimated to be eligible for ART but only 4863 were taking HAART as of March 2008 [3].

Skin manifestations constitute one of the most common clinical features in such children and their clinical pattern and severity is more or less in accordance with their CD_4_ counts. Approximately 90% of patients will develop one or more skin diseases during the course of their illness and 37% of patients present with skin lesions as a marker of HIV infection [4]. Children with HIV infection are more prone to adverse cutaneous drug reactions, both to anti-retroviral therapy (ART) and to other drugs that are given concomitantly for co-morbid illnesses. Thus, an early recognition of such features is important for an early diagnosis and also to assess the prognosis of HIV infection.

There are no data regarding prevalence and patterns of mucocutanous disorders among HIV infected children in Ethiopia. Some data from other nations are very limited and are mainly from adult cases. Extrapolating from adult cases is difficult because prevalence and pattern of mucocutanous lesions varies among children and adults. Thus, the objective of this study is to determine the prevalence and pattern of muocutanous manifestation of HIV/AIDS among children taking care and treatment in TikurAnbesa Specialized hospital (TASH).

## Methods

### Source population

All children who are followed up at the Pediatric Infectious Diseases Clinic, TikurAnbesa Specialized hospital during the study period.

### Study population

All HIV infected children who are taking care and treatment at pediatric Infectious Clinic, Tikur Anbesa Specialized Hospital during the study time. The sample size is 196 calculated using the formula *z*^*2*^*x p(1-p)/w*^*2*^, estimated prevalence of 85% (taken from research done in Tanzania) with 95% CI, and w = 0.05.

All HIV infected children aged up to 14 years, who full filled the inclusion criteria (all children living with HIV/AIDS and have been enrolled in care and treatment in Tikur anbesa hospital) and whose parents/guardians gave informed consent, were enrolled consecutively until the required sample size was obtained consecutively. HIV exposed infant where the infection was not confirmed, and children above 14 years of age who being managed on the adult clinic were excluded from the study.

### Study design

This is a descriptive cross-sectional study which was conducted at the pediatric infectious disease unit, Department of pediatrics and child health, TikurAnbesa Specialized .Hospital. In the study, both new and repeat cases were evaluated thoroughly for occurrence of mucocutanous lesions by a senior pediatric (YE) resident in collaboration with dermatologist. Data was collected using standard pretested questionnaire.

Clinical staging was done according to the WHO pediatric staging of HIV/AIDS. While immunologic classification was done according to the CDC immunologic classification for HIV infected infants and children.

Dermatological examination was done in daylight. Diagnoses of most dermatoses were done clinically. Where necessary, appropriate laboratory tests like KOH from skin scraps and gram stain, culture and sensitivity from pus swabs were performed to confirm the diagnosis.

### Data collection

All subjects had a complete history and physical examination and necessary diagnostic procedures from skin were done to confirm the diagnosis. Trained residents, nurses and data clerks working in PIDC were engaged for data collection and data entry.

### Data analysis

Statistical analysis was done using the Statistical Package for Social Sciences (SPSS) program version 16.0. Chi-squared test was used for categorical variables. A p-value of less than 0.05 was considered statistically significant.

Ethical clearance for this study was obtained from institutional research review board (IRB) of the college of health sciences. Anything that breaks the anonymity of the individual was avoided including name. Verbal assent from the child, and consent from the guardian or parent was obtained and confidentiality has been assured.

## Results

A total of 270 HIV infected children were recruited into the study. There were 48.5% (131/270) males and 51.5% (139/270) females. The age ranged from 2 months to 14 years (mean age 9.3 years with 3.5 ± SD). All were from Addis Ababa.

Over all 72.6% (196/270) had mucocutanous disorders. Almost males and females were equally infected with one or more mucocutanous lesions (72.5% and 72.6%) respectively. The prevalence of mucocutanous disorders under 5 years age was 77.7%, while for those greater than 5 years was 71.7% (p value = 0.68) (Table 1).

**Table 1:** Distribution of HIV infected children taking care and treatment by sex and age (n = 270)

**Age groups**	**Males**	**Females**	**Total**
	**n (%)**	**n (%)**	**n (%)**
0–5years	20 (15.3)	16 (11.5)	36 (13.3)
5–10 years	45 (34.3)	56 (40.2)	101 (37.4)
10–14years	66 (50.3)	67 (48.2)	133 (49.2)
Total	131 (48.5)	139 (51.5)	270 (100)

There was a wide variety of mucocutanous disorder as shown in Table 2. Infectious dermatosis accounted for the majority of mucocutanous disorders (82.6%) while noninfectious/inflammatory dermatosis accounted for 44.8% (Table 3). Many children had multiple skin conditions and were therefore counted more than once in certain situations.

**Table 2:** Mucocutanous disorders in HIV infected children attending PIDC, Tikur Anbesa Specialized Hospital (n = 196)

**Mucocutanous disorder**	**N**	**%**
**Noninfectious inflammatory dermatosis**	88	44.8
Papular pruritic rash (PPE)	60	30.6
Seborrhic dermatitis	15	7.7
Atopic dermatitis	6	3
Drug rash	6	3
Erythema nodosum	1	0.5
**Infectious dermatitis**	162	82.6
Superficial fungal infection	102	51.8
Tinea capitis	55	28
Tinea corporis	34	17.3
Tineaungum	7	3.5
Pityriasisversicolor	2	1
Oral thrush	4	2
Viral infections	38	19.3
Molluscum contagiosum	27	13.8
Herpes simplex	4	2
Herpes zoster	3	1.5
Chicken pox	3	1,5
Measles	1	0.5
Bacterial infections	20	10.2
Impetigo	18	9.2
Folliculitis	2	1
Infestation (scabies)	2	1

**Table 3:** Distribution of mucocutanous disorder in HIV infected children by age

	**Age groups**				
	**Less than 5 years**	**5–10 years**	**10–14 years**	**Total**	**p value**
**Mucocutanous disorder**	**n=28 (%)**	**n=71 (%)**	**n=97 (%)**	**n=196 (%)**	
Non infectious dermatosis	10 (35.7)	33 (46.4)	45 (46.4)	88 (44.8)	0.62
Infectious dermatosis	27 (96.4)	56 (78.8)	79 (81.4)	162 (82.6)	0.32

Superficial fungal infections were the commonest conditions present in 51.8% followed by PPE in 44.2% of the children. The most frequent superficial fungal infection was tinea capitis, occurring in 28% while the least frequent was tinea (pityriasis) versicolor found in only two children (1%). Viral and bacterial infection occurred in 19.3% and 10.2% of patients respectively. Among noninfectious dermatosis, PPE was the commonest (30.6%) followed by seborrhic dermatitis (7.7%) and the least frequent non infectious mucocutanous disorder was erythema nodosum. The commonest bacterial skin infection was impetigooccurring in 9.2%.

Molluscum contagiosum found in 13.8%, was the commonest viral infection, while measles present in only one child. Six children had drug rash induced by Nevirapine. There was no neoplasm encountered in this study.

The prevalence of mucocutaneous disorders was highest in the advanced paediatric WHO stages III (76.9%) and IV (88.8%) and relatively lower in stage II andI 61.2% and 55.5% respectively (p < 0.001). Similarly children with severe immunosuppression by CDC criteria had the highest prevalence of mucocutaneous disorders (100%), followed by those with moderate immunosuppression (70%) while children with no evidence of immunosuppression had the lowest (48.3%) prevalence (p < 0.000). The mean CD4 count was low in children who have mucocutanous lesion (456 ± 285 sd) while those who has nomucocutanous disorder was (646 ± 270 sd) with p value = 0.026 (Table 4).

**Table 4:** Prevalence of mucocutanous disorder among HIV infected children by WHO and CDC disease progression (n = 270)

	**Mucocutanous disorder**	**Mucocutanous disorder**	**Total**	**p value**
	**Present n=196**	**Absent n=74**	**n=270**	
**WHO pediatric stage**				
I	5 (55.5)	4 (44.5)	9 (3.3)	
II	63 (61.2)	40 (38.8)	103 (38.2)	< 0.001
III	80 (78)	24 (22)	104 (38.5)	
IV	48 (88.9)	6 (11.1)	54 (20)	
**Immunosuppression level by CDC**				
No evidence (category I)	44 (48.3)	47 (51.7)	91 (33.7)	
Moderate (category II)	63 (70)	27 (30)	90 (33.3)	0.000
Sever (category III)	89 (100)	0 (0)	89 (33)	
Mean cd4	456 (±285sd)	646 (±270)		0.026

Two and more mucocutanous lesions in one child were common with sever immunosuppression (34.8%) followed by moderate immunosuppression (30%) while children with no evidence of immunosuppression had lowest (22%) with p value = 0.00.

From 196 children who had mucocutanous disorders 141(72.6%) were on HAART according to Ethiopian HIV/AIDS treatment guideline. Of these 32.6% had been on HAART for more than 12 months, while 20% and 19.4% were on HAART for less than 6 months and between 6 months and 12 months respectively.

## Discussion

In this this study, 72.6% of HIV/AIDS pediatric patients attending care and treatment inTikurAnbesa Hospital had a wide range of mucocutaneous disorders. High prevalence of skin diseases among HIV infected children similar to this study has been reported in Tanzania (85%) [5], Thailand (83%) [6] and Cameroon (68.8%) [7]. Other studies showed different results [8] the difference may be due to geographic variation and quality of care. A study by Luminus et al. showed that children less than five years of age are the least affected than the older ones, [9], but in our study there is no significant difference among younger and older children similar to that of Tanzanian study [5].

According to a study in Thailand the prevalence of mucocutanous disorder in sever, moderate and no evidence of immunosuppression was 62%, 43% and 20% respectively [10] and similar study in Tanzania showed 97%, 84.5% and 71% respectively. In this study, the prevalence of mucocutaneous disorders among children with severe, moderately severe and no evidence of immunosuppression was 100%, 70% and 48.3% respectively and the differences were statistically significant (p < 0.001). Most children with moderate and sever immunosuppression have two and more mucocutanous disorder [10]. Similarly our study showed two and more skin disorders were more common in advanced immunosuppression.

All of the above studies including ours indicate that the risk of acquiring mucocutaneous disorders for HIV infected children rises as the level of immunosuppression advance. In this study Infectious dermatosis are the most frequent cause of mucocutaneous disorders among HIV infected children like other studies [5]. In this study, the most common infectious dermatosis was superficial fungal infections and the most common non infectious dermatosis was prurritic popular eruption (PPE) similar to another study [5]. The increased incidence of skin infections is attributed to the depletion of the Langerhan's cells responsible for the mucocutaneous immunological system.non infectious dermatosis also become prevalent whenthere is immunosuppression [5,7].

Patients with HIV disease are particularly prone to hypersensitivity drug eruptions [11]. In our study 6 children (3%) had moderate to severe drug rash secondary to nevirapine. kaposis sarcoma which is common cutaenous neoplasm in HIV infected adults is rare in children. In Tanzania study only one child was found to have kaposis sarcoma [5,11]. In this study there was no a case of kaposis sarcoma indicating cutanous neoplasms are rare in children.

Study done by Sibhatu B et al. on adherence to HAART in pediatric patients in Addis Ababa at different hospitals including Tikur Anbesa (Black Lion) Hospital was 86% which is low compared to other setups [12]. Some studies indicate that HAART decrease the prevalence of mucocutanous disorders especially viral infections in HIV infected individuals [13]. But in our study and Tanzanian study [5], there was no significant difference in the prevalence of mucocutanous disorder among children on HAART and pre-ART care. For ART to have a significant effect on mucocutaneous disorders, it needs to be administered for a longer period of time and patients should be adherent to treatment. In a study by Donic I. et al., it was found that the use of ART for about two years reduced significantly the presence of oral candidosis and seborrheic dermatitis [14].

This study is cross sectional which does not allow us to assess treatment response and course of mucocutanous disorder and to see effect of HAART.

## Conclusions

From this study it can be concluded that there is a high prevalence of mucocutanous disorder in HIV infected children. Most of the mucocutanous disorders were secondary to infectious causes. Children with advanced immunosuppression are suffering from a wide spectrum of mucocutanous disorders. Thus it is recommended that mucocuatnous disorders being so common among HIV infected children thorough evaluation should be done in HIV care and treatment centers to address these problems.

## Competing interests

The authors declare that they have no competing interests.

## Authors’ contributions

YTE conceived and involved in its design, data collection and write up. AWM advice, improve and supervise the study. FGD co-supervised the study and participated in the data collection. All authors read and approved the final manuscript.

## Pre-publication history

The pre-publication history for this paper can be accessed here:

http://www.biomedcentral.com/1471-5945/13/12/prepub
